# The emerging role of lncRNAs in osteoarthritis development and potential therapy

**DOI:** 10.3389/fgene.2023.1273933

**Published:** 2023-09-14

**Authors:** Xiaofeng Zhang, Qishun Liu, Jiandong Zhang, Caiyuan Song, Zongxiao Han, Jinjie Wang, Lilu Shu, Wenjun Liu, Jinlin He, Peter Wang

**Affiliations:** ^1^ Department of Traumatology, Hangzhou Fuyang Hospital of TCM Orthopedics and Traumatology, Hangzhou, Zhejiang, China; ^2^ Department of Orthopedics, Zhejiang Medical & Health Group Hangzhou Hospital, Hang Gang Hospital, Hangzhou, China; ^3^ Department of Orthopedics and Traumatology, Hangzhou Fuyang Hospital of TCM Orthopedics and Traumatology, Hangzhou, Zhejiang, China; ^4^ Zhejiang Zhongwei Medical Research Center, Department of Medicine, Hangzhou, Zhejiang, China

**Keywords:** osteoarthritis, noncoding RNA, lncRNA, biomarkers, treatment

## Abstract

Osteoarthritis impairs the functions of various joints, such as knees, hips, hands and spine, which causes pain, swelling, stiffness and reduced mobility in joints. Multiple factors, including age, joint injuries, obesity, and mechanical stress, could contribute to osteoarthritis development and progression. Evidence has demonstrated that genetics and epigenetics play a critical role in osteoarthritis initiation and progression. Noncoding RNAs (ncRNAs) have been revealed to participate in osteoarthritis development. In this review, we describe the pivotal functions and molecular mechanisms of numerous lncRNAs in osteoarthritis progression. We mention that long noncoding RNAs (lncRNAs) could be biomarkers for osteoarthritis diagnosis, prognosis and therapeutic targets. Moreover, we highlight the several compounds that alleviate osteoarthritis progression in part via targeting lncRNAs. Furthermore, we provide the future perspectives regarding the potential application of lncRNAs in diagnosis, treatment and prognosis of osteoarthritis.

## Introduction

Osteoarthritis (OA) is a common arthritis and often impairs the functions of joints, including knees, hips, hands and spine (Martel-Pelletier et al., 2016). Osteoarthritis patients exhibit various symptoms, such as pain, swelling, stiffness, and reduced mobility in joints due to the gradual breakdown and loss of cartilage in joints (Thakur et al., 2014). Osteoarthritis develops partly because of age, joint injuries, repetitive use of joints, mechanical stress, and obesity (Loeser et al., 2016; Wakale et al., 2023). In clinic, physical examination and imaging tests (X-rays and MRI) in combination with medical history are helpful for osteoarthritis diagnosis (Chalian et al., 2023; Nevalainen et al., 2023). In general, osteoarthritis was divided into four stages according to Kellgren-Lawrence classification based on radiological diagnostic criteria (Kellgren and Lawrence, 1957). Osteoarthritis patients with each stage exhibit different symptoms, joint space narrowing, cartilage loss, bone changes, and osteophyte formation (Mahmoudian et al., 2021; Szponder et al., 2022). So far, treatments mainly manage the symptoms and improve the life quality of osteoarthritis patients. Pain relievers and nonsteroidal anti-inflammatory drugs (NSAIDs) reduce pain and inflammation in osteoarthritis (Ouyang et al., 2023). For example, acetaminophen, ibuprofen, naproxen, corticosteroid injection, and hyaluronic acid injection, have been used to reduce pain and inflammation in osteoarthritis (Shentu et al., 2022; Goyal et al., 2023; Idres and Samaan, 2023; Richard et al., 2023). Exercise, weight management, and physical therapy are useful for osteoarthritis patients (Spanoudaki et al., 2023). In addition, assistive devices such as braces and canes are helpful to support joints. Joint replacement could be a useful approach for severe osteoarthritis patients (Postler et al., 2023). A healthy weight, regular exercise, and using protective techniques to protect joints can prevent and retard osteoarthritis development and progression (Mo et al., 2023; Young et al., 2023).

Genetic changes and epigenetics could involve in osteoarthritis development and progression (Hodgkinson et al., 2022; Nunez-Carro et al., 2023; Tonutti et al., 2023). For example, cellular signaling pathways have been reported to regulate osteoarthritis progression (Zhang et al., 2023a; Ruan et al., 2023; Zheng et al., 2023). In recent years, noncoding RNAs (ncRNAs) have been revealed to participate in osteoarthritis development and progression (Xie et al., 2020). It is well known that ncRNAs belong to a class of RNA molecules that do not encode proteins (Winkle et al., 2021). However, ncRNAs have diverse functions in regulation of gene expression and cellular processes (Liu and Shang, 2022). The common types of ncRNAs include microRNA (miRNA), transfer RNA (tRNA), ribosomal RNA (rRNA), long ncRNA (lncRNA), small interfering RNA (siRNA) and circular RNA (circRNA) (Eddy, 2001; Matera et al., 2007; Mattick et al., 2023). It has been shown that miRNA can bind to specific mRNA molecules and cause the inhibition of translation or degradation, leading to regulation of gene expression. LncRNAs with longer than 200 nucleotides have been identified to involve in diverse diseases via regulating gene expression and chromatin remodeling, including cancer, neurodegenerative diseases and cardiovascular disorders (Jiang et al., 2020; Cheng et al., 2022; Gareev et al., 2022; Sufianova et al., 2022; Xie et al., 2022). LncRNAs have gained significant attention and play crucial regulatory roles in osteoarthritis (Chen et al., 2017; Abbasifard et al., 2020; Tu et al., 2020; Wang et al., 2022; Okuyan and Begen, 2022). In this review, we will describe the role of numerous lncRNAs in osteoarthritis development and progression. We also mention lncRNAs as biomarkers for osteoarthritis progression. Moreover, we highlight the compounds that attenuate osteoarthritis progression via targeting lncRNAs. Furthermore, we provide the future perspectives regarding the application of lncRNAs in diagnosis, treatment and prognosis of osteoarthritis.

### Microarray and bioinformatics analyses for measuring lncRNAs in osteoarthritis

Liu et al. found that lncRNAs that are related to cartilage injury induced the degradation of chondrocyte extracellular matrix in osteoarthritis (Liu et al., 2014). By microarray and qPCR assays, this study found up to 152 lncRNAs (82 upregulated and 70 downregulated lncRNAs) to be differentially expressed between OA and normal cartilage. Depletion of cartilage injury-related lncRNAs (lncRNA-CIR) by siRNA increased collagen and aggrecan formation and decreased the expression of MMP13 and ADAMTS5, two matrix-degrading enzymes. Upregulation of lncRNA-CIR caused the opposite phenotype (Liu et al., 2014). Another group also used microarray analysis to screen the expression profile of lncRNAs in OA cartilage and normal cartilage. In this study, 73 lncRNAs were upregulated and 48 lncRNAs were downregulated in OA compared with normal cartilage (Xing et al., 2014). RT-PCR validated the six lncRNAs with upregulation in OA, including H19, CTD-2574D22.4, HOTAIR, PMS2L2, RP11-445H22.4 and GAS5. The mRNA levels of BMP-2, ADAMTS5, MMP-9, and MMP-13 were increased in OA (Xing et al., 2014). Fu and coworkers used microarray approach and provided expression profiles of lncRNAs in cartilage from knee OA patients (Fu et al., 2015). In this work, 3,007 upregulated lncRNAs and 1707 downregulated lncRNAs were discovered in OA cartilage compared with normal cartilage. Moreover, 2,136 mRNAs were upregulated and 2,241 mRNAs were downregulated in OA samples (Fu et al., 2015).

By an analysis of 19 samples from knee OA patients, 580 dysregulated lncRNAs were discovered. Four lncRNAs, SNHG5, ZFAS1, GAS5, and DANCR could be important in OA cartilage (Xiao et al., 2019). One study by Zhang et al. performed lncRNA and mRNA microarray to explore the expression files in chondrocytes from three OA patients and four healthy people in Northwest Chinese Han population (Zhang et al., 2020). Total 990 lncRNAs (660 upregulated and 324 downregulated) and 546 mRNAs (419 upregulated and 127 downregulated) were identified in OA tissues compared with normal controls. Moreover, lncRNA CTD-2184D3.4, ENST00000564198.1, and ENST00000520562.1 could control the mRNA expression levels of SPC24, GALM, and ZNF345 in OA (Zhang et al., 2020). By comprehensive analysis of mRNA and lncRNA, Luo et al. revealed different features of OA between Han population and Tibetan patients. Specifically, 117 lncRNAs (49 upregulated and 68 downregulated) and 297 mRNAs (158 upregulated and 139 downregulated) had differently expressed in the cartilage of Tibetans compared with those of Han patients (Luo et al., 2021). RNA-sequencing was performed to identify the changed expression of mRNA and lncRNAs in between 5 osteoarthritic synovium samples and 5 healthy tissues. 17 lncRNAs and 384 mRNAs were differentially expressed in OA synovium compared with healthy controls. Moreover, these differential expression of lncRNAs regulate OA progression via immune response-related pathways through lncRNA-mRNA network (Xiang et al., 2019a).

### LncRNAs regulate osteoarthritis progression

One study reported that 51 lncRNAs were upregulated and 56 lncRNAs were downregulated in the damaged cartilage tissues. LncRNA-MSR, one TMSB4 pseudogene, was elevated in the damage tissues and was upregulated in mechanical stress-stimulated chondrocytes. LncRNA-MSR competed with miRNA-152 and governed the expression of TMSB4 in chondrocytes. Hence, lncRNA-MSR upregulation enhanced cartilage degradation and initiated pathological changes (Liu et al., 2016). In the following paragraphs, we will discuss the functions and mechanisms of lncRNAs in regulation of osteoarthritis progression.

### LncRNA SNHGs

LncRNA SNHG1 (small nucleolar RNA host gene 1) has been reported to repress miR-16-5p-mediated p38 MAPK and NF-κB pathways, such as p-p65, ERK1/2 and p-p38, leading to attenuation of IL-1β-mediated osteoarthritis (Lei et al., 2019). LncRNA SNHG1 reduced the expression of several cytokines in chondrocytes, such as NO, COX-2, IL-6, PGE2, i-NOS, and THF-a. LncRNA SNHG1 also alleviated the expression of MMPs, aggrecan, collagen, and ADAMTs in chondrocytes (Lei et al., 2019). Wang et al. reported that lncRNA SNHG1 reduced cell apoptosis and inflammation via influencing the miR-195/IKK-α axis in chondrocyte (Wang et al., 2023). LncRNA SNHG5 increased proliferation of chondrocyte via targeting miR-26a/SOX2 axis in OA (Shen et al., 2018). LncRNA SNHG5 was downregulated in OA tissues. Overexpression of lncRNA SNHG5 promoted cell proliferation and migration in chondrocyte. Moreover, lncRNA SNHG5 targeted the miR-26a and then upregulated the expression of SOX2 in chondrocyte, leading to promotion of proliferation and migration of chondrocyte cells (Shen et al., 2018). Yue and coworkers found that SNHG5 blocked IL-1β-induced OA and protected chondrocytes via changing miR-181a-5p and TGFBR3 pathway (Yue et al., 2021). Jiang and colleagues observed that SNHG5 targeted miR-10a-5p and H3F3B, resulting in inhibition of apoptosis and promotion of chondrocyte proliferation in OA (Jiang et al., 2021).

LncRNA SNHG7 sponged the miR-34a-5p and upregulated SYVN1 expression, contributing to promotion of proliferation, and inhibition of apoptosis and autophagy in osteoarthritis (Tian et al., 2020). In addition, lncRNA SNHG7 attenuated miR-214-5p-mediated PPARGC1B pathway and led to inhibition of IL-1β-induced osteoarthritis via suppression of NLRP3 inflammasome and apoptosis (Xu et al., 2021). LncRNA SNHG9 suppressed cell apoptosis via downregulation of miR-34a by methylation in chondrocyte, and the expression of lncRNA SNHG9 was decreased in osteoarthritis (Zhang et al., 2020b). LncRNA SNHG12 enhanced the progression of osteoarthritis via inhibition of miR-16–5p and suppression of chondrocyte proliferation, induction of apoptosis and inflammation and ECM degradation (Yang et al., 2022). Suppression of lncRNA SNHG14 blocked FSTL-1-induced NLRP3 and TLR4/NF-kappaB pathways via regulation of miR-214–3p, conferring to inhibition of osteoarthritis (Wang et al., 2021a). LncRNA SNHG15 sponged miR-141–3p and elevated the expression of BCL2L13, resulting in prevention of osteoarthritis progression (Zhang et al., 2020). Similarly, one group found that lncRNA SNHG15 regulated ECM homeostasis and attenuated the progression of osteoarthritis (Chen et al., 2020a). Fan and colleagues reported that lncRNA SNHG16 accelerated osteoarthritis occurrence via interaction with miR-373–3p (Fan et al., 2021). In summary, lncRNA SNHGs regulate occurrence and progression of osteoarthritis (Table 1).

**TABLE 1:** LncRNA SNHGs in regulation of osteoarthritis.

LncRNA	miRNA	Targets	Functions	Ref
SNHG1	miR-16–5p	P38 MAPK, NF-κB	Attenuation of osteoarthritis	Lei et al. (2019)
SNHG1	miR-195	IKK-α	Reduces apoptosis and inflammation	Wang et al. (2023a).
SNHG5	miR-26a	SOX2	Increases proliferation of chondrocyte	Shen et al. (2018)
SNHG5	miR-181a-5p	TGFBR3	Blocks IL-1β-induced osteoarthritis	Yue et al. (2021)
SNHG5	miR-10a-5p	H3F3B	Inhibition of apoptosis and promotion of proliferation	Jiang et al. (2021)
SNHG7	miR-34a-5p	SYVN1	Promotes proliferation, inhibits apoptosis and autophagy	Tian et al. (2020)
SNHG7	miR-214–5p	PPARGC1B	Inhibits IL-1β-induced osteoarthritis	Xu et al. (2021)
SNHG9	miR-34a	N/A	Suppresses cell apoptosis	Zhang et al. (2020b)
SNHG12	miR-16–5p	N/A	Induces apoptosis, inflammation, ECM degradation, inhibits proliferation	Yang et al. (2022a)
SNHG14	miR-214–3p	FSTL-1, NLRP3, NF-κB	Inhibits osteoarthritis	Wang et al. (2021a)
SNHG15	miR-141–3p	BCL2L13	Prevents osteoarthritis progression	Zhang et al. (2020c)
SNHG16	miR-373–3p	N/A	Accelerates osteoarthritis occurrence	Fan et al. (2021a)

### LncRNA HOTAIR

LncRNA HOTAIR (HOX transcript antisense RNA) promoted ADAMTS-5 expression in osteoarthritic articular chondrocytes (Dou et al., 2017). LncRNA HOTAIR was highly expressed in human OA cartilage. TNF-α (tumor necrosis factor) increased the expression of HOTAIR in OA chondrocytes. Knockdown of HOTAIR reduced the expression of ADAMTS-5, while upregulation of HOTAIR elevated ADAMTS-5 expression levels in OA chondrocytes. HOTAIR regulated the ADAMTS-5 mRNA stability in OA articular chondrocytes (Dou et al., 2017). Hu et al. found that HOTAIR facilitated osteoarthritis progression via regulation of miR-17–5p, fucosyltransferase 2 (FUT2) and β-catenin (Hu et al., 2018). Higher expression of HOTAIR was linked to chondrocyte apoptosis, ECM degradation and modified Mankin scale. HOTAIR interacted with miR-17–5p and upregulated FUT2 expression, leading to regulating wnt/β-catenin pathway. Moreover, HOTAIR aggravated chondrocyte apoptosis and ECM degradation (Hu et al., 2018). Overexpression of HOTAIR induced apoptosis of chondrocytes and caused IL-1β-mediated MMP upregulation in temporomandibular joint OA (Zhang et al., 2016). It is recognized that lncRNA HOTAIR-involved Wnt/β-catenin pathway regulated the pathogenesis of cartilage damage via modulation of MMP-13 (Zhou et al., 2019). Upregulation of HOTAIR induced an increase in apoptosis rates and reduced the viability of chondrocytes via sponging miR-130a-3p to inhibit autophagy in chondrocytes in knee OA, which was accompanied by the downregulation of Bcl-2 and survivin and upregulation of cleavage of caspase 3 and Bax expression (He and Jiang, 2020). Chen et al. reported that HOTAIR accelerated OA progression via sponging miR-20b and upregulating PTEN (Chen et al., 2020b).

**FIGURE 1:**
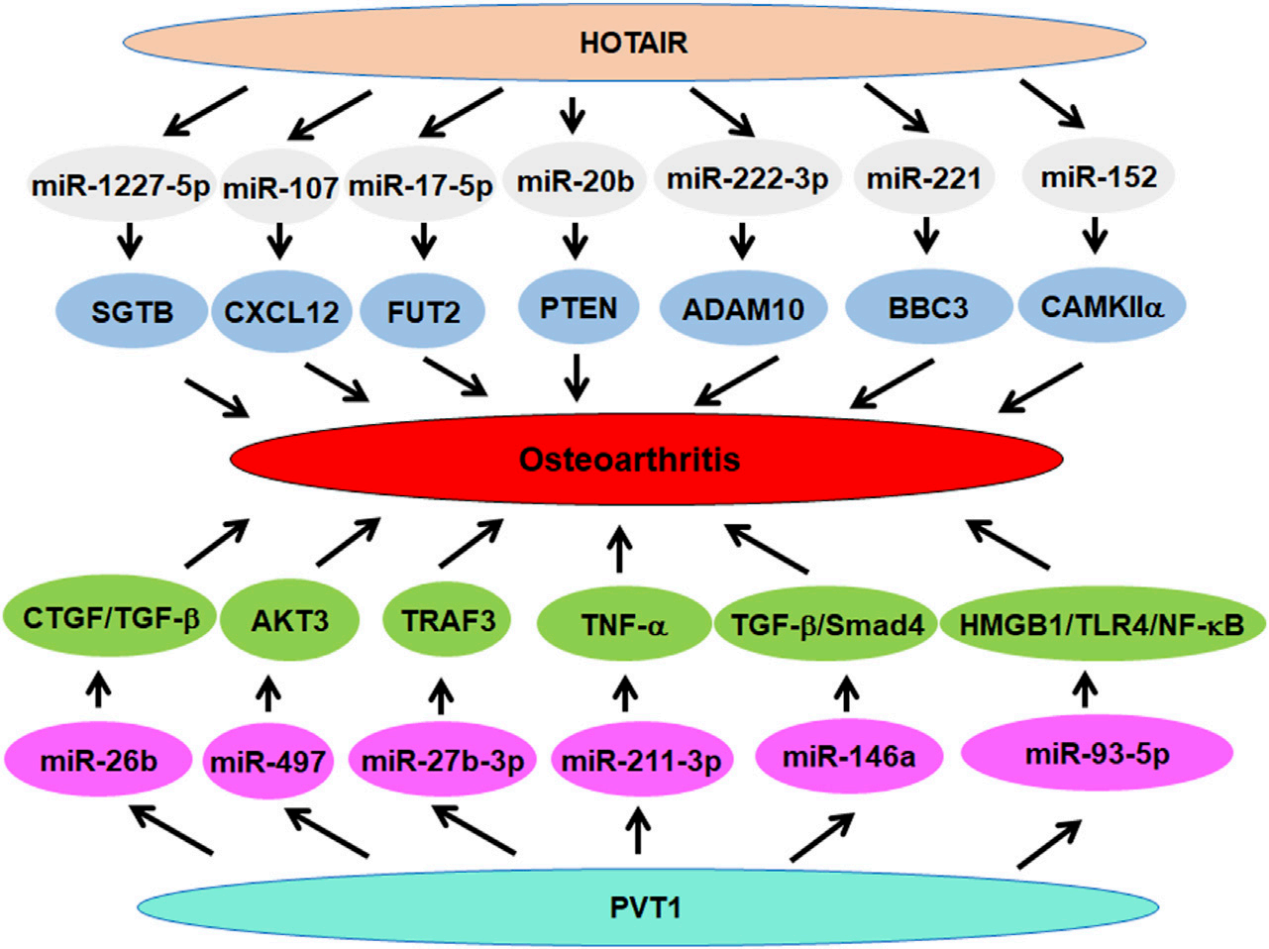
The role of lncRNA HOTAIR and lncRNA PVT1 in osteoarthritis.

HOTAIR promoted cartilage degradation by suppression of Wnt inhibitory factor 1 (WIF-1) expression via enhancing histone H3K27 trimethylation in WIF-1 promoter, resulting in activation of the Wnt/β-catenin pathway in OA (Yang et al., 2020). Osteopontin induced the expression of HOTAIR in the primary chondrocytes. Overexpression of HOTAIR and osteopontin promoted chondrocyte proliferation, whereas downregulation of HOTAIR reduced proliferation of chondrocyte cells, suggesting that HOTAIR could involve in OA via influencing cell proliferation (Liang et al., 2021). HOTAIR induced inflammation and LPS-induced chondrocyte apoptosis through influencing miR-1227–5p and increasing the expression of small glutamine rich tetratricopeptide repeat containing beta (SGTB) in OA (Wang et al., 2021b). Lu et al. observed that knockdown of HOTAIR blocked OA chondrocyte injury via modulation of miR-107 and CXCL12 pathway (Lu et al., 2021). IL-1β-mediated cell apoptosis, oxidative stress, inflammatory response, and ECM degradation were blocked by inhibition of HOTAIR in chondrocytes by affecting miR-222–3p and ADAM10 axis (Wang et al., 2021). Similarly, HOTAIR promoted mechanical stimulation-mediated apoptosis via regulation of miR-221 and BBC3 pathway (Zheng et al., 2021). In addition, dysregulation of HOTAIR in craniosynostosis led to impaired osteoclast differentiation via changing miR-152-CAMKIIα pathway (Dong et al., 2022). HOTAIR expression was positively associated with TNF-α, hs-CRP, IgA, total cholesterol (TC), and VAS (visual analog scale) score (Chen et al., 2023). Upregulation of HOTAIR repressed cell proliferation, increased the expression of TNF-α, p-PI3K, and p-AKT, attenuated PTEN and IL-10 expression in OA chondrocytes after stimulation by OA PBMCs (peripheral blood mononuclear cells) (Chen et al., 2023). Altogether, HOTAIR plays an essential role in OA progression (Figure 1).

### LncRNA PVT1

LncRNA PVT1 expression was higher in OA chondrocytes than that in normal chondrocytes. Downregulation of PVT1 reduced the apoptosis of OA chondrocytes, while normal chondrocytes increased apoptosis after PVT1 upregulation. PVT1 induced apoptosis via sponging miR-488–3p in OA (Li et al., 2017). Zhao et al. found that PVT1 depletion abolished the suppression of IL-1β on aggrecan and collagen II expression, and reduced IL-1β-mediated induction of MMPs, such as MMP-3, MMP-9 and MMP-13. PVT1 downregulation abrogated the IL-1β-stimulated production of inflammatory cytokines, such as PGE2, TNF-α, IL-6, NO, and IL-8. Molecular mechanism revealed that PVT1 sponged miR-149 to perform regulate metabolic dysfunction in OA chondrocytes (Zhao et al., 2018). PVT1 regulated hyperglycemia-mediated collagen degradation via sponge of miR-26b and regulation of CTGF/TGF-β signaling pathway (Ding et al., 2020). In line with this study, PVT1 sponged miR-140 and induced chondrocyte ECM degradation in IL-1β-treated chondrocytes (Yao et al., 2022). One study showed that depletion of PVT1 blocked IL-1β-mediated injury by targeting miR-27b-3p and TRAF3 in chondrocytes (Lu et al., 2020).

Another study reported that PVT1 upregulated TNF-α in synoviocytes and increased apoptosis of chondrocyte via sponge of miR-211–3p (Xu et al., 2020). Similarly, depletion of exosome-mediated lncRNA PVT1 regulated the HMGB1/TLR4/NF-κB pathway by sponging miR-93–5p, resulting in LPS-mediated OA progression (Meng et al., 2020). In addition, PVT1 inhibited miR-146a and activated TGF-β/SMAD4 signaling, leading to promotion of cartilage degradation in diabetic OA mice (Wang et al., 2021). LncRNA PVT1 and GAS5 (growth arrest specific 5) regulated each other to govern chondrocyte apoptosis in osteoarthritis (Cai et al., 2022). PVT1 upregulation induced apoptosis, while GAS5 upregulation reduced apoptosis in chondrocytes induced by LPS, indicating that PVT1 and GAS5 have a negative feedback loop (Cai et al., 2022). Recently, PVT1 regulated miR-497/AKT3 pathway and influenced the function of osteoarthritis cells via regulation of proliferation, apoptosis and ECM degradation in chondrocytes (Xu J. et al., 2022). Taken together, PVT1 participates in the development and progression of osteoarthritis (Figure 1).

### LncRNA MEG3

**FIGURE 2:**
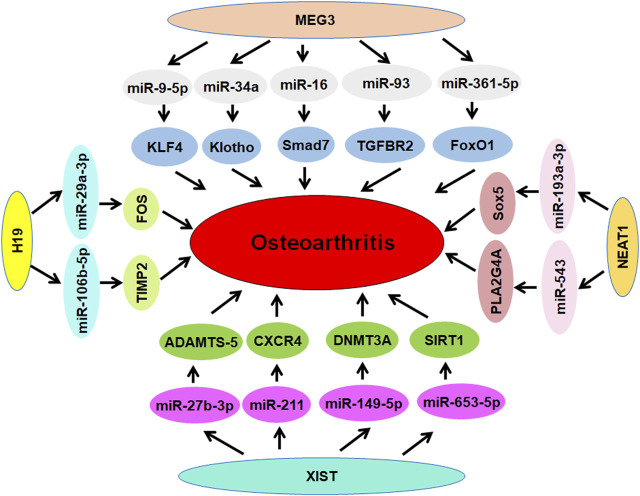
The role of lncRNAs MEG3, H19, NEAT1, XIST in osteoarthritis.

The expression of lncRNA maternally expressed gene 3 (MEG3) was decreased in patients with osteoarthritis compared with healthy cartilage samples by real-time RT-PCR. The expression of lncRNA MEG3 was inversely correlated with VEGF levels, which was measured by ELISA in cartilage tissues. This study indicated that lncRNA MEG3 could participate in osteoarthritis development via the modulation of angiogenesis (Su et al., 2015). Similarly, one group also found that lncRNA MEG3 was downregulated in rat osteoarthritis cartilage tissues. Inhibition of MEG3 enhanced proliferation and reduced apoptosis in IL-1β-mediated rat chondrocytes. MEG3 inhibited the expression of miR-16 and elevated SMAD7 expression in IL-1β-treated chondrocytes. Hence, inhibition of MEG3 could cause osteoarthritis progression via targeting miR-16/SMAD7 axis (Xu and Xu, 2017). Methylene blue, an inhibitor of peripheral nerve axons to alleviate pain, has been shown to increase the expression of lncRNA MEG3 and decrease P2X purinoceptor 3 (P2X3) protein levels. Methylene blue increased the expression of IL-6, IL-8, IL-1β and TNFα. Methylene blue could relieve the inflammation and pain via promotion of lncRNA MEG3 in osteoarthritis (Li et al., 2018a). Chen et al. reported that lncRNA MEG3 attenuated the ECM degradation of chondrocytes and induced proliferation and inhibited apoptosis of chondrocytes through modulation of miR-93 and TGFBR2 pathways in osteoarthritis (Chen et al., 2021). In line with this report, lncRNA MEG3 reduced apoptosis and induced proliferation of chondrocytes via influencing miR-361–5p/FoxO1 axis in osteoarthritis (Wang et al., 2019). Notably, lncRNA MEG3 retarded chondrogenic differentiation of synovium-derived mesenchymal stem cells (SMSCs) via suppression of TRIB2 by methyltransferase EZH2 (You et al., 2019). LncRNA MEG3 reduced chondrocyte impairment by IL-1β-mediated inflammation due to governing miR-9-5p/KLF4 axis (Huang et al., 2021). In addition, one report showed that lncRNA MEG3 controlled osteoarthritis progression via affecting miR-34a/Klotho axis (Xiong et al., 2022). LncRNA MEG3 is critically involved in osteoarthritis development and progression (Figure 2).

### LncRNA H19

Several studies have demonstrated that lncRNA H19 regulates osteoarthritis progression. For example, depletion of lncRNA H19 reduced LPS-mediated damage via regulation of miR-130a in osteoarthritis (Hu et al., 2019). LncRNA H19 exhibited the diagnostic value in the blood of osteoarthritis (Zhou et al., 2020). Peripheral blood of osteoarthritis patients had an increased expression of lncRNA H19, which was linked to the occurrence and development of osteoarthritis (Zhou et al., 2020). LncRNA H19 expression was elevated in osteoarthritis samples. Overexpression of lncRNA H19 enhanced apoptosis and decreased the proliferation in IL-1β-mediated chondrocytes via sponging miR-106a-5p in osteoarthritis (Zhang et al., 2019). LncRNA H19 regulated cartilage matrix degradation and calcification via interaction with miR-140–5p in osteoarthritis (Yang et al., 2020). Exosomal lncRNA H19 regulated the progression of osteoarthritis via governing the miR-106b-5p/TIMP2 axis (Tan et al., 2020). In addition, lncRNA H19 gene polymorphisms were associated with risk of osteoarthritis in a Chinese Han population (Wang et al., 2020). Umbilical cord blood MSCs produced lncRNA H19 controlled central sensitization of pain via targeting miR-29a-3p/FOS pathway in osteoarthritis (Yang et al., 2021). LncRNA H19 relieved inflammation via influencing TP53, IL-38, and IL-36 receptors in osteoarthritis (Zhou et al., 2022). Motion-mediated posttraumatic osteoarthritis was abrogated by moderate-intensity treadmill running due to upregulation of lncRNA H19 expression (Zhou. et al., 2021). Hence, lncRNA H19 influences osteoarthritis progression (Figure 2).

### LncRNA XIST

LncRNA XIST was highly expressed in cartilage tissues of osteoarthritis patients. Downregulation of lncRNA XIST inhibited IL-1β-suppressed proliferation and induced apoptosis in chondrocytes. XIST can sponge miR-211 and upregulate the expression of CXCR4, leading to modulation of chondrocyte proliferation and apoptosis via targeting MAPK signaling pathway (Li et al., 2018). Methylation of TIMP-3 promoter was accelerated by lncRNA XIST, contributing to collagen degradation in osteoarthritic chondrocytes in patients with tibial plateau fracture (Chen et al., 2019). Sun et al. reported that miR-142–5p prevented osteoarthritis progression due to the interaction between lncRNA XIST and miR-142–5p (Sun et al., 2020). Similarly, lncRNA XIST protected chondrocytes from IL-1β-mediated injury through sponging miR-653–5p and upregulating SIRT1 (Lian and Xi, 2020). In line with this finding, lncRNA XIST conferred to osteoarthritis development by influencing miR-149–5p and DNMT3A pathways (Liu et al., 2020a). Chondrogenic differentiation of SMSCs was governed by lncRNA XIST through interaction with miR-27b-3p and upregulation of ADAMTS-5 (Zhu et al., 2021). In addition, YY1 increased the expression of lncRNA XIST, resulting in suppression of cartilage differentiation of BMSCs via interacting with TAF15 to maintain the stability of FUT1 protein (He et al., 2022). Interestingly, lncRNA XIST interacted with miR-150–5p and affected monocyte adherence, suggesting that lncRNA XIST might be useful for osteoarthritis treatment (Wang et al., 2022). In conclusion, lncRNA XIST regulates progression of osteoarthritis (Figure 2).

### LncRNA MALAT1

**FIGURE 3:**
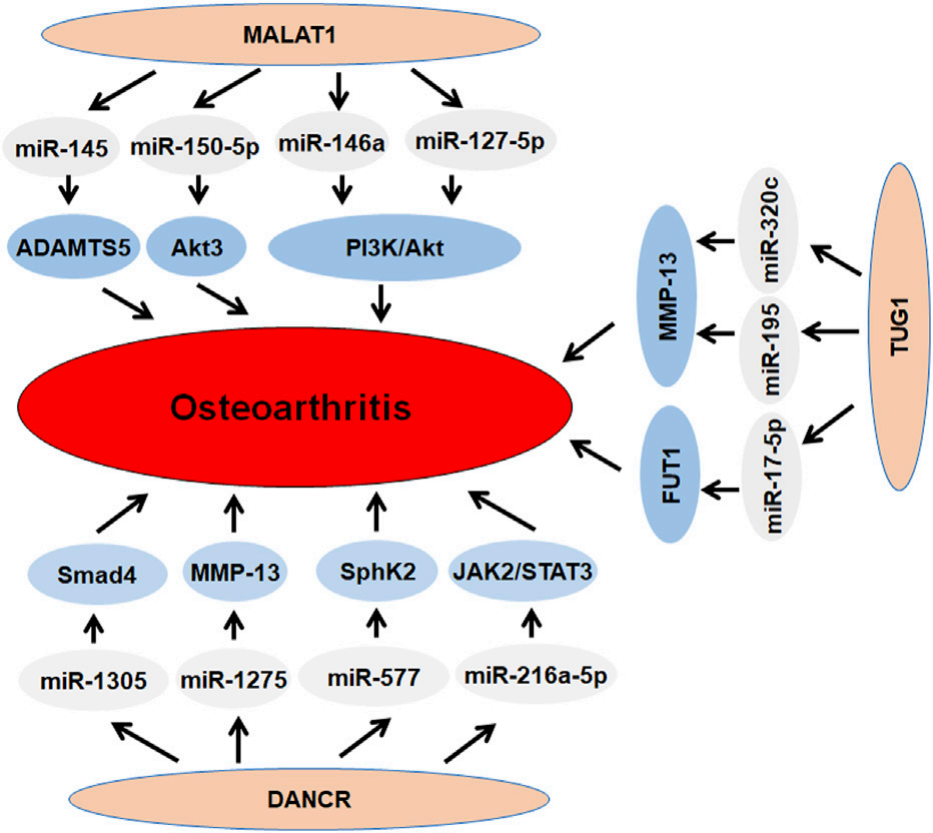
The role of lncRNAs MALAT1, TUG1, DANCR in osteoarthritis.

LncRNA MALAT1 regulated PI3K/AKT pathway via targeting miR-127–5p and controlled osteopontin (OPN)-induced cell proliferation in human chondrocytes (Liang et al., 2018). MALAT1 targeted the JNK pathway and reduced apoptosis and matrix metabolism disorder in articular chondrocytes with IL-β-mediated inflammation (Gao et al., 2019). Zhang et al. found that MALAT1 influenced miR-150–5p and AKT3 pathways and accelerated osteoarthritis development (Zhang et al., 2019). Li et al. reported that MALAT1 sponged miR-146a and modulated PI3K/Akt/mTOR pathway, leading to regulation of chondrocyte proliferation after LPS treatment (Li et al., 2020). Liu and coworkers observed that MALAT1 sponged miR-145 and elevated ADAMTS5, resulting in regulation of IL-β-mediated viability and cartilage matrix degradation in osteoarthritis (Liu et al., 2019). MALAT1 was found to regulate the inflammatory synovial fibroblast phenotype in obese patients with osteoarthritis (Nanus et al., 2020). One group validated the expression and function of MALAT1 in osteoblasts from osteoarthritis patients (Alnajjar et al., 2021). MALAT1 from MSCs-derived extracellular vesicles blocked cartilage degradation and attenuated inflammation in osteoarthritis (Pan et al., 2021). Another group reported that miR-124–3p impaired MALAT1 stability and led to suppression of chondrocyte pytoptosis and inhibition of cartilage injury in osteoarthritis (Rozi et al., 2022). Altogether, MALAT1 controls osteoarthritis development and progression (Figure 3).

### LncRNA NEAT1

LncRNA NEAT1 regulated cartilage matrix degradation via targeting miR-193a-3p and SOX5 pathways in osteoarthritis (Liu F. et al., 2020). Inhibition of lncRNA NEAT1 reduced apoptosis, and inflammation, and extracellular matrix degradation, while inhibiting the expression of MMP-3, MMP-13, and ADAMTS-5. Additionally, it increased Col2a1 and ACAN expression in chondrocytes (Liu et al., 2020). One group showed that lncRNA NEAT1 increased chondrocyte proliferation and reduced apoptosis via inhibition of miR-16–5p in osteoarthritis (Li. et al., 2020). Another group reported that lncRNA NEAT1 governed miR-543/PLA2G4A axis and led to inhibition of chondrocyte proliferation and induction of apoptosis in osteoarthritis (Xiao et al., 2021). Overexpression of lncRNA NEAT1 alleviated the expression of Bcl-2 and p-Akt1, elevated the expression of MMP-3, MMP-9, MMP-13, IL-6 and IL-8 in chondrocytes (Xiao et al., 2021). Wang et al. reported that NEAT1 sponged miR-378 and regulated LPS-mediated articular chondrocytes, resulting in influencing the osteoarthritis development (Wang et al., 2022). Moreover, achyranthes bidentata polysaccharides (ABPS) increased the expression of lncRNA NEAT1 and decreased the miR-377–3p expression, leading to attenuation of endoplasmic reticulum in osteoarthritis (Fu et al., 2022). Therefore, lncRNA NEAT1 affects osteoarthritis development (Figure 2).

### LncRNA DANCR

LncRNA DANCR has been reported to promote proliferation and differentiation of chondrogenesis of synovium-derived mesenchymal stem cells (SMSCs) (Zhang et al., 2017a). LncRNA DANCR upregulation increased proliferation and chondrogenesis of SMSCs via interaction with myc, Smad3, and STAT3 mRNA to influence their stability. DANCR increased the expression of Smad3 and STAT3, leading to activation of chondrogenesis of SMSCs (Zhang et al., 2017a). SOX4 was found to promote chondrogenic differentiation and proliferation of SMSCs by upregulation of lncRNA DANCR (Zhang et al., 2015). Moreover, lncRNA DANCR modulated miR-1305/Smad4 axis to enhance chondrogenic differentiation of SMSCs (Zhang et al., 2017b). Furthermore, DANCR accelerated chondrogenesis via modulation of miR-1275 and MMP-13 in SMSCs (Fang et al., 2019). LncRNA DANCR sponged miR-577 and elevated SphK2 expression, contributing to promotion of proliferation of chondrocytes and induction of apoptosis in osteoarthritis development (Fan et al., 2018). Moreover, lncRNA DANCR influenced chondrocyte proliferation and apoptosis through regulation of miR-216a-5p, JAK2 and STAT3 pathways in osteoarthritis (Zhang et al., 2018). These findings indicate that lncRNA DANCR plays an essential role in osteoarthritis progression (Figure 3).

### LncRNA TUG1

LncRNA taurine upregulated gene 1 (TUG1) has been found to be elevated in cartilages of osteoarthritis patients compared with normal cartilages. IL-1β and TNF-α induced the expression of lncRNA TUG1 in chondrocytes. Upregulation of lncRNA TUG1 suppressed the miR-195 expression and inhibited the expression of collagen and aggrecan, whereas lncRNA TUG1 overexpression promoted the expression of MMP-13, indicating that TUG1 might promote degradation of chondrocyte extracellular matrix in osteoarthritis by regulation of miR-195 and MMP-13 (Tang et al., 2018). Li et al. found that depletion of lncRNA TUG1 reduced the expression of MMP-13 and induced the expression of collagen II and aggrecan in IL-1β-treated chondrocyte. Moreover, TUG1 targeted miR-17–5p and elevated the expression of fucosyltransferase 1 (FUT1). Silencing of TUG1 repressed osteoarthritis progression via downregulation of FUT1 by inhibition of miR-17–5p, which was due to promotion of viability and inhibition of apoptosis and ECM degradation in chondrocytes (Li et al., 2020c). Duan et al. reported that LncRNA TUG1 levels and loci at rs5749201, rs7284767 and rs886471 were correlated with knee osteoarthritis development (Duan et al., 2021). In addition, one study revealed that lncRNA TUG1 governed ECM degradation of chondrocytes in osteoarthritis via control of miR-320c/MMP-13 pathway (Han and Liu, 2021). Taken together, lncRNA TUG1 could be associated with osteoarthritis pathogenesis (Figure 3).

### Other lncRNAs

Accumulating evidence indicated that besides the abovementioned lncRNAs, numerous other lncRNAs regulate osteoarthritis development and progression (Table 2). For instance, lncRNA AC006064.4-201 destabilized CDKN1B mRNA by interaction with PTBP1, which led to alleviation of cartilage senescence and protection of osteoarthritis (Shen et al., 2023). LncRNA WDR11-AS1 can bind to RNA-binding protein PABPC1 and then increase the SOX9 stabilization, resulting in facilitating extracellular matrix synthesis in osteoarthritis (Huang et al., 2023). LncRNA NAV2-AS5 alleviated chondrocyte inflammation via inhibition of miR-8082 and upregulation of TNFAIP3 interacting protein 2 (TNIP2) in osteoarthritis (Wang et al., 2023). LncRNA TM1-3P affected miR-144–3p and ONECUT2 and influenced apoptosis, inflammation, and proliferation of fibroblasts in osteoarthritis (Yi et al., 2022). LncRNA PMS2L2 suppressed proliferation of chondrocyte via promotion of miR-34a expression in osteoarthritis (Yang et al., 2022). LncRNA MINCR blocked progression of osteoarthritis through upregulation of BMPR2 expression by sponging miR-146a-5p (Li et al., 2022). LncRNA CRNDE relieved the progression of osteoarthritis via upregulation of dapper antagonist of catenin-1 (DACT1) by epigenetic modification and inactivation of Wnt/β-catenin pathway (Zhang et al., 2022). LncRNA Gm37494 relieved chondrocyte injury by interacting with miR-181a-5p and upregulating GABRA1 expression in osteoarthritis (Yuan et al., 2022). LncRNA LEMD1-AS1 alleviated chondrocyte inflammation by upregulation of PGAP1 via sponging miR-944 in osteoarthritis (Li et al., 2022).

**TABLE 2:** Numerous lncRNAs regulate osteoarthritis progression.

LncRNA	miRNA	Targets	Functions	Ref
NAV2-AS5	miR-8082	TNIP2	Alleviates chondrocyte inflammation	Wang et al. (2023b)
TM1-3P	miR-144–3p	ONECUT2	Influences apoptosis, inflammation and proliferation	Yi et al. (2022)
PMS2L2	miR-34a	N/A	Suppresses proliferation of chondrocyte	Yang et al. (2022b)
MINCR	miR-146a-5p	BMPR2	Blocks progression of osteoarthritis	Li et al. (2022a)
Gm37494	miR-181a-5p	GABRA1	Relieves chondrocyte injury	Yuan et al. (2022)
LEMD1-AS1	miR-944	PGAP1	Alleviates chondrocyte inflammation	Li et al. (2022b)
POU3F3	miR-29a-3p	FOXO3	Relieves osteoarthritis pathogenesis	Shi et al. (2022)
MCM3AP-AS1	miR-149–5p	Notch1	Promotes osteoarthritis progression	Xu et al. (2022b)
LINC00473	miR-424–5p	LY6E	Exacerbates osteoarthritis progression, induces apoptosis, proinflammatory cytokine production	Fan et al. (2021b)
LINC01385	miR-140–3p	TLR4	Promotes osteoarthritis progression	Wang et al. (2021e)
MIR22HG	miR-9-3p	ADAMTS5	Facilitates osteoarthritis	Long et al. (2021)
CASC19	miR-152–3p	DDX6	Enhances apoptosis, induces proinflammatory cytokine production	Zhou et al. (2021b)
FGD5-AS1	miR-302d-3p	TGFBR2	Inhibits osteoarthritis development	Yang et al. (2021b)
KCNQ1OT1	miR-211–5p	TCF4	Promotes osteoarthritis	Aili et al. (2021)
OIP5-AS1	miR-29b-3p	PGRN	Aggravates osteoarthritis progression	Zhi et al. (2021)
HOTTIP	miR-455–3p	CCL3	Causes cartilage degradation	Mao et al. (2019)
HOTTIP	miR-663a	Fyn	Accelerates osteoarthritis	He et al. (2021b)
RMRP	miR-206	CDK9	Induces apoptosis, inhibits proliferation	Lu et al. (2020b)
LOXL1-AS1	miR-423–5p	KDM5C	Enhances osteoarthritis	Chen et al. (2020c)
LINC00511	miR-150–5p	SP1	Affects proliferation and apoptosis	Zhang et al. (2020d)
IGHCγ1	miR-6891–3p	TLR4	Regulates macrophage inflammation	Zhang et al. (2020e)
LINC00623	miR-101	HRAS	Regulates senescence and apoptosis	Lu et al. (2020c)
NKILA	miR-145	SP1, NF-kB	Inhibits apoptosis, induces proliferation	Xue et al. (2020)
MFI2-AS1	miR-130a-3p	TCF4	Promotes LPS-induced osteoarthritis	Luo et al. (2020)
PART1	miR-373–3p	SOX4	Impacts proliferation and apoptosis	Zhu and Jiang (2019)
PART1	miR-590–3p	TGFBR2, Smad3	Regulates viability, apoptosis	Lu et al. (2019a)
TNFSF10	miR-376–3p	FGFR1	Governs osteoarthritis progression	Huang et al. (2019a)
KLF3-AS1	miR-206	GIT1	Regulates proliferation and apoptosis	Liu et al. (2018)
FOXD2-AS1	miR-206	CCND1	Controls proliferation of chondrocytes	Cao et al. (2018a)

LncRNA ZNFX1 antisense 1 (ZFAS1) reduced anti-oxidative stress via sponge of miR-1323 in osteoarthritis (Gu et al., 2022). LncRNA PILA enhanced the activity of PRMT1 and activated NF-κB pathway in osteoarthritis (Tang et al., 2022). LncRNA PRNCR1 affected apoptosis and proliferation of synoviocytes via binding with miR-377–3p in osteoarthritis (Wang G. et al., 2022). LncRNA POU3F3 relieved osteoarthritis pathogenesis via interaction with miR-29a-3p and upregulation of FOXO3 (Shi et al., 2022). LncRNA MCM3AP-AS1 promoted progression of osteoarthritis via modulation of miR-149–5p and Notch1 pathways (Xu et al., 2022). LINC00265 sponged miR-101–3p and regulated proliferation, inflammation and apoptosis of chondrocytes in osteoarthritis (Zou et al., 2021). LINC00473 exacerbated progression of osteoarthritis by enhancement of proinflammatory cytokine production and induction of chondrocyte apoptosis via influencing the miR-424–5p/LY6E axis (Fan et al., 2021). LINC01385 downregulation abolished progression of osteoarthritis via sponging miR-140–3p and increasing TLR4 expression (Wang Z. et al., 2021). LncRNA THUMPD3-AS1 accelerated inflammatory response and promoted chondrocyte proliferation in osteoarthritis (Wang et al., 2021). Downregulation of HOTAIRM1-1 promoted osteoarthritis development via regulation of miR-125b in cartilage tissues (Liu et al., 2021). LncRNA MIR22HG facilitated osteoarthritis progression via targeting miR-9-3p/ADAMTS5 axis (Long et al., 2021). LncRNA FER1L4 governed the expression of IL-6 and regulated osteoarthritis in chondrocyte cells (He et al., 2021). LncRNA CASC19 exacerbated osteoarthritis development via modulation of miR-152–3p and DDX6, which enhanced chondrocytes apoptosis and induced proinflammatory cytokine production (Zhou et al., 2021).

LncRNA FGD5-AS1 controlled miR-302d-3p and TGFBR2 expression and caused inhibition of osteoarthritis development (Yang et al., 2021). Silencing of lncRNA KCNQ1OT1 blocked the progression of osteoarthritis via changing the miR-211–5p/TCF4 axis (Aili et al., 2021). LncRNA OIP5-AS1 sponged the miR-30a-5p and affected the function of chondrocytes in osteoarthritis (Qin et al., 2021). Similarly, another study found that depletion of OIP5-AS1 affected miR-29b-3p/PGRN axis and aggravated osteoarthritis development (Zhi et al., 2021). LncRNA HOTTIP sponged miR-455–3p and increased the expression of CCL3 and led to degradation of cartilage (Mao et al., 2019). Moreover, HOTTIP was reported to control miR-663a/Fyn-related kinase axis and accelerate osteoarthritis progression (He et al., 2021). Depletion of lncRNA RMRP repressed apoptosis and induced proliferation of osteoarthritis chondrocytes via inhibition of miR-206 and upregulation of CDK9 (Lu et al., 2020). LncRNA PCAT-1 modulated the expression of miR-27b-3p and changed apoptosis of chondrocytes in osteoarthritis (Zhou et al., 2021). LncRNA MIR4435-2HG suppressed the expression of miR-510–3p and attenuated osteoarthritis progression (Liu et al., 2020c). Chen et al. found that lncRNA LOXL1-AS1 modulated the miR-423–5p/KDM5C pathway and resulted in osteoarthritis progression, which could be activated by JUND (Chen et al., 2020). Zhang et al. found a positive feedback loop between LINC00511, miR-150–5p and SP1, which affected chondrocyte apoptosis and proliferation in osteoarthritis (Zhang et al., 2020). LncRNA IGHCγ1 reduced the expression of miR-6891–3p and upregulated TLR4 expression levels, contributing to regulation of macrophage inflammation in osteoarthritis (Zhang et al., 2020). LncRNA CTBP1-AS2 promoted the miR-130a gene methylation and led to suppression of chondrocyte proliferation in osteoarthritis (Zhang et al., 2020f). LncRNA BLACAT1 was discovered to govern differentiation of bone marrow stromal stem cells via sponging miR-142–5p in osteoarthritis (Ji et al., 2020). IL-1β-induced degradation of ECM was modulated by LINC00623 due to regulation of miR-101 and HRAS, leading to regulation of senescence and apoptosis of osteoarthritis chondrocytes (Lu et al., 2020). Downregulation of lncRNA NKILA enhanced apoptosis and reduced proliferation of chondrocytes by targeting miR-145, SP1 and NF-κB in osteoarthritis (Xue et al., 2020).

LncRNA CASC2 can be regulated by miR-93–5p and affect chondrocyte apoptosis in osteoarthritis (Sun et al., 2020). Li and coworkers found that lncRNA ANRIL regulated apoptosis and proliferation of osteoarthritis synoviocytes and governed osteoarthritis progression (Li et al., 2019). Downregulation of lncRNA MFI2-AS1 reduced LPS-mediated osteoarthritis progression via impacting the miR-130a-3p/TCF4 axis (Luo et al., 2020). LncRNA CAIF attenuated LPS-mediated IL-6 upregulation via suppression of miR-1246 in osteoarthritis (Qi et al., 2019). LncRNA PART1 impacted miR-373–3p/SOX4 axis and affected proliferation and apoptosis of chondrocytes as well as ECM degradation in osteoarthritis (Zhu and Jiang, 2019). LncRNA PART1 sponged miR-590–3p and regulated TGFBR2/Smad3, resulting in regulation of viability and apoptosis of chondrocytes in osteoarthritis (Lu et al., 2019). LncRNA MIR4435-2HG was dissected to regulate proliferation and apoptosis of chondrocyte in osteoarthritis (Xiao et al., 2019). LncRNA TNFSF10 impacted the miR-376–3p/FGFR1 pathway and led to osteoarthritis progression (Huang et al., 2019). One positive association between lncRNA ANCR and TGF-β1 was reported in osteoarthritis patients (Li et al., 2019). LncRNA DILC was identified to regulate the expression of IL-6 in chondrocytes and participated in osteoarthritis (Huang et al., 2019). Similarly, lncRNA CASC2 regulated the expression of IL-17 and modulated apoptosis and proliferation of chondrocytes in osteoarthritis (Huang et al., 2019). LncRNA FOXD2-AS1 facilitated proliferation of chondrocytes via suppression of miR-27a-3p in osteoarthritis (Wang et al., 2019b). LncRNA TM1P3 mediated ECM degradation of chondrocytes and took part in osteoarthritis progression (Li et al., 2019). LINC00341 blocked osteoarthritis progression via enhancement of chondrocyte survival (Yang et al., 2019). Inhibition of LOC101928134 reduced the synovial hyperplasia and cartilage degradation of osteoarthritis rats via activation of JAK/STAT pathway by promotion of IFNA1 expression (Yang et al., 2019).

Lu et al. reported that cell apoptosis of chondrocytes was governed by lncRNA-CIR in osteoarthritis (Lu et al., 2019). LncRNA-CIR modulated autophagy and accelerated degeneration of articular cartilage in osteoarthritis (Wang et al., 2018). Moreover, lncRNA CIR sponged miR-27b and accelerated ECM degradation of chondrocytes in osteoarthritis (Li et al., 2017). LncRNA KLF3-AS1 affected miR-206 and GIT1 expressions in osteoarthritis, contributing to MSC-derived exosome-mediated promotion of proliferation and inhibition of apoptosis of chondrocytes (Liu et al., 2018). LncRNA-p21 served as a sponge of miR-451 and led to induction of apoptosis of chondrocytes in osteoarthritis (Tang et al., 2018). LncRNA GACAT3 governed IL-6/STAT3 pathway and induced proliferation of synoviocytes in osteoarthritis (Li et al., 2018c). LncRNA FOXD2-AS1 sponged miR-206 and upregulated CCND1 expression, governing proliferation of chondrocyte in osteoarthritis (Cao et al., 2018a). LncRNA FAS-AS1 was dissected to accelerate the ECM degradation of cartilage in osteoarthritis (Zhu et al., 2018). One study identified the critical role of lncRNA ZFAS1 in regulation of migration, apoptosis and proliferation of chondrocytes in osteoarthritis (Ye et al., 2018). Depletion of lncRNA RP11-445H22.4 reduced LPS-mediated injuries via modulation of miR-301a in osteoarthritis (Sun et al., 2018). LncRNA UFC1 facilitated proliferation of chondrocytes due to sponging miR-34a in osteoarthritis (Zhang et al., 2016). Taken together, lncRNAs play an essential role in osteoarthritis progression.

### LncRNAs as biomarkers for osteoarthritis progression

Wu et al. identified exosomal mRNA, lncRNA and circRNA signatures in an OA synovial fluid-exosomal study (Wu et al., 2022). This work reported that 196 lncRNAs, 98 circRNAs, and 52 mRNAs were differentially expressed between healthy control and OA synovial exosomes. Moreover, 45 lncRNAs, 34 circRNAs, and 22 miRNAs were associated with the PI3K/Akt and autophagy pathways, which were linked to 7 mRNAs and might contribute to OA pathological process (Wu et al., 2022). One group identified that the expression of exosomal lncRNA PCGEM1 was higher in early OA than normal controls, and higher in late-stage OA than in early OA, suggesting that synovial fluid-derived exosomal lncRNA PCGEM1 could be a useful biomarker for the different stages of OA (Zhao and Xu, 2018). LncRNA Nespas was reported to be associated with osteoarthritis pathogenesis by upregulation of ACSL6, indicating that Nespas could act as a prognostic biomarker (Park et al., 2019). LncRNA HOTTIP was upregulated in the processes of endochondral ossification and osteoarthritis pathogenesis. HOTTIP might be a potential predictive biomarker for osteoarthritis (Kim et al., 2013). In addition, lncRNA DANCR was elevated in osteoarthritis patients and was validated as a useful biomarker and treatment target for osteoarthritis (Zhang et al., 2018). LncRNA TNFSF10 was validated as a novel potential biomarker for osteoarthritis progression (Huang et al., 2019). One study suggested that lncRNA SNHG5 might be a promising biomarker for osteoarthritis treatment (Jiang et al., 2021). Another study used RNA sequencing and found LINC00167 as a novel diagnosis biomarker for osteoarthritis (Jiang et al., 2020). LncRNA HOTAIR was highly expressed in osteoarthritis chondrocytes, suggesting that it could be a biomarker for osteoarthritis (Chen et al., 2023). Altogether, lncRNAs could act as biomarkers for osteoarthritis diagnosis and prognosis.

### Compounds target lncRNAs to regulate osteoarthritis progression

In recent years, some compounds have been identified to alleviate osteoarthritis progression (Wu et al., 2023). Baicalin, a flavonoid isolated from the roots of *Scutellaria baicalensis* Georgi (Lamiaceae), has been primarily used in traditional Chinese Medicine (Srinivas, 2010). Baicalin has been reported to treat different diseases via exerting its various functions, such as anticancer, antioxidant, antiviral, anti-inflammatory effects (Li et al., 2021; Ganguly et al., 2022; Song et al., 2023; Wang and Li, 2023). Baicalin has shown its protective functions in OA pathological process. For example, baicalin prevented endplate chondrocyte apoptosis via suppression of H_2_O_2_-mediated oxidative stress (Pan et al., 2017). Baicalin reduced IL-1β-induced inflammatory response in human chondrocytes (Xing et al., 2017). Baicalin inhibited the expression of miR-126 and reduced IL-1β-triggered inflammatory injury in chondrocytes (Yang et al., 2018). In line with this report, Baicalin reduced muscular oxidative stress and alleviated joint pain and muscle dysfunction in OA rat model (Chen et al., 2018). One study showed that Baicalin modulated endoplasmic reticulum stress and protected chondrocytes from OA (Cao et al., 2018b). Another study demonstrated that baicalin blocked IL-1β-stimulated apoptosis and ECM degradation via activation of autophagy by targeting miR-766–3p and AIFM1 pathway, leading to protection of OA chondrocytes (Li et al., 2020d). Baicalin activated HIF-1α and increased extracellular matrix synthesis in chondrocytes (Wang et al., 2020). Moreover, baicalin enhanced the extracellular matrix synthesis and elevated chondrocyte viability via modulation of TGF-β/Smad3 pathway in chondrocytes (Wang et al., 2021). Recently, baicalin alleviated IL-1β-mediated OA chondrocytes damage via promotion of mitophagy (He and He, 2023). Notably, baicalin exerted therapeutic effects by inhibiting the expression of lncRNA HOTAIR, decreasing the protein levels of p-PI3K and p-AKT, and increasing the protein levels of PTEN, APN, and ADIPOR1 (Chen et al., 2023). Hence, baicalin could be a useful agent to protect OA.

Schisantherin A attenuated IL-1β-mediated inflammation via inactivation of NF-κB and MAPKs in chondrocytes (Liao et al., 2016). One study showed that protectin DX repressed IL-1β-involved inflammation and ameliorated osteoarthritis development via regulation of the AMPK and NF-κB pathways in chondrocytes (Piao et al., 2020). Salvianolic acid B reduced IL-1β-mediated inflammatory cytokine production in chondrocytes in osteoarthritis and protected osteoarthritis progression (Lou et al., 2017). Daurisoline activated the PI3K/Akt/mTOR axis and led to inhibition of H2O2-mediated autophagy in chondrocytes (Zhang et al., 2023b). Oroxin B suppressed the PI3K/Akt/mTOR pathway and induced autophagy and anti-inflammation, leading to inhibition of osteoarthritis progression (Lu et al., 2022). Icariin, a kind of flavonoid compound, promoted HIF-1α in chondrocytes and accelerated cartilage repair (Wang et al., 2016). Resveratrol was reported to regulate the lncRNA MALAT1 and miR-9/NF-κB axis and retard osteoarthritis progression (Zhang et al., 2020). Kaempferol reduced the functions of lncRNA XIST and miR-130a/STAT3 on ECM degradation and inflammation in osteoarthritis (Xiao et al., 2021). Hence, compounds could attenuate osteoarthritis progression via targeting lncRNA expressions.

## Conclusion and perspectives

In summary, numerous lncRNAs play a critical regulatory role in osteoarthritis development and progression. Multiple lncRNAs could be useful for acting biomarkers for diagnosis, prognosis and therapeutic targets. Further understanding the functions and molecular mechanism of lncRNAs in osteoarthritis is necessary to improve the therapeutic outcome of osteoarthritis patients. Without a doubt, several issues should be mentioned to better understand the role of ncRNAs in osteoarthritis. Besides lncRNAs, miRNAs, and circRNAs have also been reported to participate in osteoarthritis progression. It is known that circRNAs form a covalently closed loop and regulate gene expression, leading to governing cellular processes. Accumulating evidence has suggested that circRNAs play a critical role in osteoarthritis initiation and progression (Wu and Zou, 2021; Wang et al., 2023; Li and Lu, 2023; Xue et al., 2023).

Xiang et al. used RNA sequencing and revealed the circular RNA expression profiles in osteoarthritic synovium (Xiang et al., 2019b). By an integrated bioinformatics analysis, 120 circRNAs were differentially expressed in OA synovium. Five decreased circRNAs and one increased circRNAs were confirmed by qRT-PCR approach (Xiang et al., 2019b). One study identified circRNA expression profile of articular chondrocytes using IL-1β-induced osteoarthritis in mice (Zhou et al., 2018). Another study screened differentially expressed circRNAs of cartilages in patients with osteoarthritis (Wang et al., 2019c). In addition, lncRNAs are involved in rheumatoid arthritis development (Lao and Xu, 2020). For example, lncRNA SNHG1 interacted with PTBP1 (polypyridine tract-binding protein 1) and promoted rheumatoid synovial proliferation and invasion (Liu F. et al., 2021). One lncRNA can regulate the expression of the other lncRNA to regulate osteoarthritis. For instance, lncRNA PACER overexpression suppressed apoptosis of chondrocyte. PACER upregulation led to inhibition of HOTAIR. HOTAIR upregulation induced chondrocyte apoptosis. Consistently, plasma PACER was decreased in osteoarthritis patients, whereas plasma HOTAIR was increased in OA samples. PACER governed chondrocyte apoptosis via inhibition of HOTAIR in osteoarthritis (Jiang et al., 2019).

In this review, we summarize the functions and mechanisms of numerous lncRNAs in governing OA pathogenesis. It is unclear which lncRNA plays the most important role in regulating OA development and progression. Which lncRNAs are key biomarkers for diagnosis and prognosis of OA patients? Which strategy is a best approach for targeting lncRNAs in OA treatment? It is necessary to explore whether anti-inflammatory drugs in combination with lncRNA inhibitors or activators would bring a better benefit for OA patients. Altogether, in-depth investigations are required to determine the molecular mechanisms of lncRNAs-mediated osteoarthritis development and progression.
